# Estimating the Effect of Reduced Attendance at Emergency Departments for Suspected Cardiac Conditions on Cardiac Mortality During the COVID-19 Pandemic

**DOI:** 10.1161/CIRCOUTCOMES.120.007085

**Published:** 2020-12-20

**Authors:** Michail Katsoulis, Manuel Gomes, Alvina G. Lai, Albert Henry, Spiros Denaxas, Pagona Lagiou, Vahe Nafilyan, Ben Humberstone, Amitava Banerjee, Harry Hemingway, R. Thomas Lumbers

**Affiliations:** 1 Institute of Health Informatics (M.K., A.G.L., A.H., S.D., A.B., H.H., R.T.L.), University College London, United Kingdom.; 2 Health Data Research UK London (M.K., A.G.L., A.H., S.D., A.B., H.H., R.T.L.), University College London, United Kingdom.; 3 Institute of Epidemiology & Health Care (M.G.), University College London, United Kingdom.; 4 The National Institute for Health Research University College London Hospitals Biomedical Research Centre (S.D., H.H., R.T.L), University College London, United Kingdom.; 5 British Heart Foundation Research Accelerator (S.D.), University College London, United Kingdom.; 6 The Alan Turing Institute, London, United Kingdom (S.D.).; 7 Department of Hygiene, Epidemiology and Medical Statistics, School of Medicine, National and Kapodistrian University of Athens, Greece (P.L.).; 8 Department of Epidemiology, Harvard T.H. Chan School of Public Health, Boston, MA (P.L.).; 9 Office for National Statistics, Newport, United Kingdom (V.N., B.H.).; 10 Amrita Institute of Medical Sciences, Kochi, India (A.B.).; 11 Bart’s Heart Centre, St. Bartholomew’s Hospital, London, United Kingdom (R.T.L.).

**Keywords:** cardiovascular diseases, coronavirus, death, sudden, cardiac, heart disease, pandemic

The wider health impacts of the coronavirus disease 2019 (COVID-19) pandemic are of increasing concern, with an increase in rates of non-COVID-19 excess mortality observed. In England and the United States, the early pandemic was accompanied by a decline in patient visits to Emergency Departments (EDs), including those for cardiac diseases.^1,2^ The decline may have been influenced by patient’s reluctance to visit hospital due to the public health messages to protect National Health Service capacity, concerns about the risk of coronavirus infection, or difficulties in accessing medical care. The impact of delayed or nonpresentation to EDs with suspected cardiac disease on cardiac mortality is unknown. In this study, we used instrumental variable analysis to estimate the effects of reduced ED visits on cardiac mortality in England.

To quantify the change in daily ED visits for suspected cardiac disease, we extracted data from the Public Health England Emergency Department Syndromic Surveillance System weekly bulletins, a sentinel network of EDs across England using Web Plot Digitizer (https://automeris.io/WebPlotDigitizer). We additionally obtained weekly mortality counts for cardiac disease from the Office of National Statistics for England. We estimated daily mortality counts from the weekly data by assuming a linear trend. Cardiac mortality was defined as death due to coronary heart disease, heart failure, or sudden cardiac death (*International Classification of Diseases*-Tenth *Revision* codes used to classify these outcomes are available online on the HDR UK Phenotype ID portal: https://portal.caliberresearch.org/phenotypes/katsoulis-noncovid19cvd-death-a69hszdh3dcgzpn4hjcnkg). We excluded deaths where COVID-19 was included in the registration. To enable comparison with mortality data for England, we scaled the daily Emergency Department Syndromic Surveillance System visit data up by a factor of 3; since, Emergency Department Syndromic Surveillance System^1^ covers 60 of a total of 180 EDs in England.^3^ Data on mortality outcomes are available from the Office of National statistics upon request and ED attendance data are available online (https://www.gov.uk/government/publications/syndromic-surveillance-weekly-summaries-for-2020). The analysis script is available upon request to Michail Katsoulis (m.katsoulis@ucl.ac.uk). This study used population-level data for ED visits and mortality and was performed in accordance with guidelines for study procedures provided by the UCL Research Ethics Committee.

To explore how the reduction of suspected cardiac disease affected cardiac mortality, we utilized an instrumented difference-in-differences design, using the COVID-19 pandemic as the instrument.^4,5^ To define the level of the instrumental variable, we selected March 12, the date when the UK Chief Medical Officers raised the UK risk from COVID-19 to high, following the WHO declaration of a global pandemic (March 11). We estimated the relationship between daily ED visits and cardiac deaths by using the 2-stage least squares method.^5^ First, we regressed the exposure (ED visits for cardiac diseases) on the instrument (COVID-19 pandemic), assigning the value of 0 if the ED visit occurred before March 12, 2020, or 1 if on or after this date. Second, we regressed the outcome (number of cardiac deaths) on the predicted exposure. In both steps, the following 4 terms were included to adjust for seasonality^5^: (1) period (0 for previous years, 1 for this year [December 18, 2019 to April 15, 2020]), (2) t (time in days from 12/18), (3) squared t, and (4) cubic t. The mortality associated with untreated acute cardiac disease may occur immediately or after a delay, or lag period, the length of which is determined by the type and severity of the presenting disease. For example, untreated myocardial infarction may result in early arrhythmic death or later death due to heart failure. We, therefore, estimated the effects of reduced ED visits on cardiac deaths for a range of time lag periods (delays), between nonpresentation and the associated mortality, from 0 to 20 days.

During the COVID-19 pandemic period (December 12, 2020 to April 15, 2020), we estimated a decline of 2750 (95% CI, 2504–2996) ED visits per week for suspected cardiac disease (≈35% decrease compared with the average weekly admission before the pandemic this year). We estimated that every 100 non-attendances at EDs for suspected cardiac disease were associated with between 3.1 (95% CI, 1.5–4.6) and 8.4 (95% CI, 7.0–9.8) excess cardiac deaths, corresponding to the lower and upper bound of the estimated mortality lag times of 0 and 18 days, respectively. The weekly excess cardiac mortality during the pandemic period, due to nonattendance at EDs, was estimated to be between 84 (95% CI, 42–127) and 232 (95% CI, 193–270) deaths (see Figure). This corresponds to an increase in weekly non-COVID-19 cardiac mortality of up to 18%, compared with the previous 5 years, and implies that one cardiac death could have been prevented or delayed for every 12 ED visits with suspected cardiac disease. These estimates were robust to sensitivity analysis whereby we altered the date used to define the COVID19 pandemic period by 3 days, before and after March 12.

**Figure.**
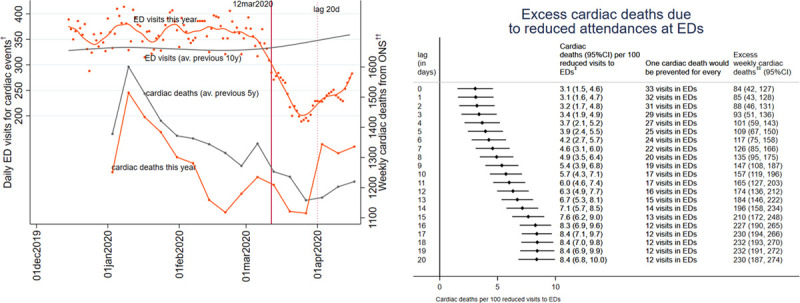
**Left: observed number of attendances in emergency department (EDs) for suspected cardiac events (left *y* axis) in England from Emergency Department Syndromic Surveillance System (EDSSS), compared with the average of the previous 10 y and N of cardiac deaths in England, compared with the average of the previous 5 y from the Office of National Statistics.** Right: estimated effect of reduced emergency department (ED)† attendance for suspected cardiac events on cardiac deaths, in England, during the coronavirus disease 2019 (COVID-19) pandemic, using instrumented difference-in-difference. To define the level of the instrumental variable, we selected March 12, 2020 as the first day of the pandemic. Estimates are given for a range of mortality lag times, the time between the reduced ED attendance for suspected cardiac conditions and cardiac deaths, from 0 to 20 d. ONS indicates Office of National Statistics. †Data provided from Public Health England from 60 EDs that are part of EDSSS (out of the 180 in England). ††Data provided from Office of National Statistics for England. ‡Cardiac deaths caused per 100 reduced ED attendances for suspected cardiac events. ‡‡Excess weekly cardiac deaths due to reduced ED attendance for suspected cardiac events.

In our study, we use modern causal inference methodology^4,5^ to estimate to what extent cardiac mortality was affected by reduced attendances in EDs for cardiac conditions. It is likely that premature non-COVID-19 mortality, due to undertreatment of acute cardiac disease, will continue to accumulate beyond the 20-day follow-up period attainable at the time of this study. The validity of our estimates rests upon the assumption that the COVID-19 pandemic only affects excess deaths from cardiac disease through the reduction in ED admissions (exclusion restriction assumption), and not through other factors, such as, increased anxiety leading to an increased incidence rate of myocardial infarction. Another potential limitation of this study is the possible misclassification of CVD deaths as not related to COVID-19, since severe acute respiratory syndrome coronavirus 2 (SARS-CoV-2) viral infection was excluded on clinical grounds rather than through systematic viral RNA testing. Finally, this study was based on attendance data for a sample of 60, from a total of 180 EDs, in England; we were unable to assess the representativeness of this sample and cannot exclude the possibility that our estimates differ from the true effects due to biased sampling.

We found evidence of reduced ED attendances of patients with suspected cardiac disease during the COVID19 pandemic peak in England and an associated time-lagged increase in cardiac mortality. We estimate the unintended harm that may have resulted from the effect of the COVID19 pandemic response on health care-seeking behavior and/or the provision of health care for non-COVID19 cardiac disease. Our study was limited to evaluating the short-term mortality associated with reduced ED presentations; however, there is a risk that longer term impacts of undertreatment on cardiovascular morbidity and mortality will continue to emerge over time. These findings should alert policymakers to the importance of ensuring that any measures introduced to control and manage SARS-CoV-2 infection do not adversely impact the management of acute cardiovascular disease.

## Sources of Funding

Dr Katsoulis is funded by the British Heart Foundation (grant: FS/18/5/33319). Dr Denaxas is supported by an Alan Turing Fellowship. Dr Lai is funded by the Wellcome Trust (204841/Z/16/Z), the National Institute for Health Research (NIHR) Great Ormond Street Hospital Biomedical Research Centre (19RX02) and the NIHR University College London Hospitals Biomedical Research Centre (BRC714a/HI/RW/101440). Dr Hemingway is a NIHR Senior Investigator. His work is supported by (1) Health Data Research UK (grant No. LOND1), which is funded by the UK Medical Research Council, Engineering and Physical Sciences Research Council, Economic and Social Research Council, Department of Health and Social Care (England), Chief Scientist Office of the Scottish Government Health and Social Care Directorates, Health and Social Care Research and Development Division (Welsh Government), Public Health Agency (Northern Ireland), British Heart Foundation and Wellcome Trust. (2) The BigData@Heart Consortium, funded by the Innovative Medicines Initiative-2 Joint Undertaking under grant agreement No. 116074. This Joint Undertaking receives support from the European Union’s Horizon 2020 research and innovation programme and EFPIA; it is chaired, by DE Grobbee and SD Anker, partnering with 20 academic and industry partners and ESC. (3) The National Institute for Health Research University College London Hospitals Biomedical Research Centre. Dr Lumbers is supported by a UK Research and Innovation Rutherford Fellowship hosted by Health Data Research UK (MR/S003754/1), the BigData@Heart Consortium funded by the Innovative Medicines Initiative-2 Joint Undertaking under grant agreement No. 116074, and the National Institute for Health Research University College London Hospitals Biomedical Research Centre.

## Disclosures

None.
